# Postharvest CO_2_ treatment and cold storage for *Drosophila suzukii* (Diptera: Drosophilidae) fruit infestation control

**DOI:** 10.1093/jee/toae264

**Published:** 2024-11-15

**Authors:** Nika Cvelbar Weber, Špela Modic, Primož Žigon, Jaka Razinger

**Affiliations:** Department of Fruit Growing, Viticulture and Oenology, Agricultural Institute of Slovenia, Ljubljana, Slovenia; Plant Protection Department, Agricultural Institute of Slovenia, Ljubljana, Slovenia; Plant Protection Department, Agricultural Institute of Slovenia, Ljubljana, Slovenia; Plant Protection Department, Agricultural Institute of Slovenia, Ljubljana, Slovenia

**Keywords:** integrated pest management, fruit infestation, fruit sanitation, post-harvest treatment, spotted wing drosophila

## Abstract

The invasive pest, spotted wing drosophila (*Drosophila suzukii* (Matsumura, 1931) or SWD), damages various soft-skinned fruits, severely impacting orchards and vineyards economically. Current sorting practices in commercial production may overlook early-stage SWD infestations, as visible signs take a few days to appear. Our study focused on managing SWD infesting fruits (blueberry, cherry, and raspberry) without visible signs using an artificial atmosphere with elevated CO_2_ and low temperature. We hypothesized that these factors affect SWD survival and possibly interact, with potential variations among different soft- or stone-fruit species or varieties. High CO_2_ concentrations and cold storage both negatively affected SWD development. A 24-h 100% CO_2_ fumigation, without cold storage, significantly reduced SWD infestations in all 3 fruit species studied. On the other hand, 10% CO_2_ without cold storage did not cause a significant infestation reduction in cherries. Cold storage alone was too slow to be considered effective. Concurrent low-temperature treatment and CO_2_ treatment reduced the insecticidal efficacy of CO_2_ fumigation. Optimal fruit sanitation was achieved with a 3-h 100% CO_2_ treatment at ambient temperature before cold storage. Raspberries were the most suitable host for SWD development, with over a 5-fold higher SWD development compared to blueberries and over 50 times more than in cherries. We discussed the observed interactions between CO_2_ fumigation and chilling and suggested a simple postharvest SWD management protocol using optimal CO_2_ levels, exposure times, and chilling periods—achievable without complex equipment.

## Introduction

Spotted wing Drosophila (SWD), or the *Drosophila suzukii* [Matsumura, 1931], (Diptera: Drosophilidae) is an invasive, polyphagous pest that infests a wide range of wild and domestic soft-skinned fruits and causes economic damage, especially in orchards and vineyards (Walsh et al. 2011, Winkler et al. 2020, de Groot et al. 2022). It originates from Southeast Asia and is spreading in North and South America as well as in Europe (Cini et al. 2014, Mendonca et al. 2019). In Slovenia, SWD was confirmed in October 2010 but was probably present earlier and has spread rapidly throughout the country (Seljak 2011). This small polyphagous fruit fly has a rapid dispersal ability, high reproductive potential, and the ability to adapt to a wide range of climatic conditions (Cini et al. 2012), which poses a challenge for integrated pest management control strategy. In contrast to the other *Drosophila* species in Europe, the SWD females have a hardened and serrated ovipositor (Lee et al. 2011), which enables them to carry out an initial infestation at the time of fruit ripening and, in the case of ripe fruit, shortly before harvest. Also, infestations after harvest in storage facilities are reported. Significant economic damage was reported in 2008 where the damage in just 3 US states was estimated at 511.3 million dollars (Bolda et al. 2010). The registered insecticides pyrethroids, organophosphates, spinosyns, ryanoids, and neonicotinoids from various classes have primarily been used to control SWD based on monitoring of adult activity (Beers et al. 2011, Bruck et al. 2011, Sial et al. 2019). Presently, the possibility of using effective insecticides is limited due to the short pre-harvest interval, residual activity, inadequate relative efficacy, and zero tolerance policy in the fresh fruit market (Stark and Banks 2003, Van Timmeren and Isaacs 2013). Therefore, to reduce damage in the field and after harvest, different management strategies for pest control are important. Besides insecticides, other non-chemical approaches have also been investigated in practice to reduce the use of insecticides, such as behavior-based management strategies (Rice et al. 2017), biological control (Cini et al. 2012, Lee et al. 2019), as well as cultural and management tactics (Haye et al. 2015, Diepenbrock et al. 2017, Leach et al. 2018, Stockton et al. 2019).

Soft fruit and cherries popularity among consumers is increasing due to their organoleptic and health-promoting properties (Manganaris et al. 2014). Nevertheless, the inherent high perishability of these fruits presents a notable challenge in ensuring an extended shelf life for them. As they belong to a group of non-climacteric fruits, the senescence of the fruit begins immediately after harvest. Therefore, the postharvest conditions and treatments are crucial to maintain fruit quality from the field through storage to the point of sale. Various methods have been proposed to prevent post-harvest decay and prolong the shelf life of fresh blueberries, cherries and raspberries. Their shelf life in regular cold storage at 0.5–4°C varies between 14 and 20 d, depending on pre-harvest factors (i.e., production technology, plant species, cultivar, ripening stage at harvest, harvesting method) (Matiacevich et al. 2013). There are many ways to further adjust cold storage with the goal to slow down respiration and thus fruit aging for a longer period (Horvitz 2017). Effective preservation of the quality of soft fruit and cherries can be achieved by a combination of low temperatures and a change in the gas composition in the storage atmosphere (Terry et al. 2009). The maintenance of fruit quality for non-climacteric fruits was successfully achieved with an exposure to elevated carbon dioxide (CO_2_) levels. Subsequent to this treatment, the fruits are stored under standard cold storage conditions at 1–4°C for an additional 11 d, proving to be an effective preservation method (González-Orozco et al. 2020). High levels of CO_2_ not only decrease susceptibility to fungal attacks but also play a role in mitigating respiration, minimizing water loss, and impeding the softening process in fruit berries. Combined impact of low temperature and modified atmosphere serves as an effective means for extending the postharvest life of soft fruit and cherries (Horvitz 2017).

Cooling and modifying the atmosphere during storage are also common physical pest control methods that are used as postharvest strategies for soft fruit (Aly et al. 2017) and cherries (Mostafa et al. 2021). The use of cold storage is a regular part of an integrated program for table grape producers to control various fruit fly species such as *Ceratitis capitata* and *Bactrocera tryoni* (De Lima et al. 2011) and has also been proposed to manage SWD infestations (Aly et al. 2017, Kraft et al. 2020). The SWD is widely distributed in temperate climates and is considered a cold-sensitive species (Jakobs et al. 2015). Exposure to low temperatures causes various physiological disturbances, such as protein denaturation and cellular depolarization due to loss of ionic balance (Enriquez and Colinet 2019, Tarapacki et al. 2021). Previous studies investigating the effects of postharvest cold storage temperatures and exposure durations on survival and development of immature SWD have shown that postharvest cold storage temperatures and exposure durations reduce survival and prolong development time (Aly et al. 2017, Kraft et al. 2020). In addition to temperatures, atmospheric changes in cold storage that reduce oxygen levels combined with an increase in CO_2_ levels or the addition of other fumigants, are recommended measures to control immature stages of SWD (Jeon et al. 2022, Seok et al. 2022, Chen et al. 2023). Reduced O_2_ (hypoxia) and/or increased CO_2_ (hypercapnia) can affect the respiration rate of insects and thus the rate and biochemistry of metabolism.(Boardman et al. 2011, Cao et al. 2019) Higher concentrations of CO_2_ could potentially impact the physiological processes of SWD, although the specific effects would depend on the concentration and duration of exposure. Acute exposure to CO_2_ can lead to a decrease in performance in *Drosophila* larvae, including continuous respiration resulting in increased gas exchange that can cause uncontrolled water loss leading to dehydration, acidification of blood and tissue fluids, denaturation of enzymes, and a subsequent decrease in glutathione production (Badre et al. 2005).

The usual storage conditions for extending the shelf life of soft fruit require an increased CO_2_ concentration between 10% and 20% and cold storage at an average temperature of 1–4°C (depending on the fruit species) (Yang et al. 2009, Choi et al. 2013). Commercial production practice requires harvested fruits to be transported to a sorting facility. There, fruits with visible malformations, like softened tissue after SWD infestation, are removed. However, SWD infestation is not evident in the first 2 to 3 d and thus often goes unnoticed during sorting (Sial 2022). Consequently, SWD-infested fruit often proceeds to the packaging line and further toward sale and, on the shelf, SWD can continue to develop. Our study therefore specifically targeted fruits that do not yet exhibit visual signs of SWD infestation and cannot be identified using conventional methods in the postharvest chain. We hypothesized that artificial atmosphere with elevated CO_2_ concentrations and low temperature will affect the survival of SWD, infesting soft fruits and cherries. We also theorized there are interactions between the two factors and that different soft-fruit species or varieties will be differentially susceptible to SWD infestation. Therefore, the objectives of the current study were to (i) investigate the possible use of elevated CO_2_ concentrations and low temperature treatment as postharvest strategy to control SWD in artificially infested blueberries, raspberries, and cherries, (ii) to determine what is the optimal exposure time of lab-reared SWD to elevated CO_2_ atmosphere prior to cold storage, to obtain a strong CO_2_ fumigation effect before cold storage, (iii) to verify the results of aims (i) and (ii) on naturally infested fruit, and (iv) to assess the suitability of different fruit species or varieties for SWD development.

## Materials and Methods

### *Drosophila suzukii* Rearing and Artificial Fruit Infestation

The SWD flies of different ages used in the experiments were from a laboratory colony reared on an artificial diet, as described previously (Razinger et al. 2017). In brief, the SWD were reared in 30 × 30 × 30 cm plastic insectaria (BugDorm-1, BugDorm, Taiwan) in a growth chamber in dark:light cycles of 14:10 h at 21°C and 77 ± 3% relative humidity. The flies were provided with tap water and solid SWD artificial food medium (20 g agar, 20 g sugar, 10 g wheat flour, 50 g dry baker’s yeast, 500 ml tap water, 400 g grated organic apples, 500 ml organic apple juice, 50 ml apple vinegar, and 4 g nipagin) (methyl 4-hydroxybenzoate, Sigma-Aldrich, Germany). The culture was established in 2017. Since then, no wild flies were added to the colony.

Organically produced blueberries (*Vaccinium corymbosum* cv. ‘Bluecrop’ and ‘Eliot’), cherries (Prunus avium cv. ‘Kordia’), and raspberries (*Rubus idaeus* cv. ‘Amira’) were harvested from the experimental orchard of the Agricultural Institute of Slovenia in Brdo pri Lukovici (46°10ʹ02.4″ N, 14°40ʹ48.9″ E). The fruits were harvested at technological maturity and transported to the laboratory on the same day. After harvesting, 1.5 kg of fruit was placed in a large insectarium (60 × 60 × 90 cm; Entosphinx, Czech Republic) and exposed to a population of SWD flies of different ages and mixed sexes to infest the fruit. The fruit was exposed to SWD flies for 48 h to ensure thorough infestation, under the same temperature and humidity conditions as when the SWD colony was maintained (21°C, 77 ± 3% RH 14: 10 (L:D) h).

#### Producing Artificial Atmospheres

After exposure to SWD, the infested fruits were removed from the infestation insectariums and randomly divided into smaller samples of 15–30 blueberries (ca. 20–30 g), 15 cherries (ca. 200 g), or 6 raspberries (ca. 30 g). One such sample was considered a biological replicate (see below for detailed description of specific experiments). The infested fruit samples were transferred to plastic containers (insect breeding dish, square, 72 × 72 × 100 mm, HiMedia, India) with ventilation holes and nylon netting on the lid to allow air circulation. The plastic containers containing infested fruit were packed in polyethylene vacuum bags. The bags were sealed airtight and the entire atmosphere was removed using an air sealer (Besser Vacuum srl, Smart, Dignano, Italy). A modified atmosphere was injected directly into the sealed polyethylene vacuum bags.

#### Experiments Assessing Elevated CO_2_ Concentration and Short-term Cold Storage as a Combined Effect

To assess the effect of different storage conditions on the development of SWD, samples were exposed to standard postharvest practice for soft fruit (Horvitz 2017) with elevated CO_2_ concentration (10%), reduced O_2_ concentration (5%) while the rest (85%) was N_2_. We also exposed the samples to 100% CO_2_ concentration following the same procedure. Both treatments (i.e., 10% and 100% CO_2_) were compared to treatment with normal atmosphere (i.e., 0.4% CO_2_). All treatments were supplemented with cold storage (4.0 ± 0.5°C, relative humidity 90%) for 0 h (treatments assessing only CO_2_-fumigation effect), 24, 48, or 72 h. Thus, 12 different treatments were made (3 CO_2_ concentrations and 4 levels of cold storage). Samples (i.e., infested fruit packed in vacuum sealed plastic bags) from treatments with 0 and 24 h of cold storage were kept in their particular atmospheres for 24 h, whereas samples from treatments with 48 and 72 h of cold storage were kept in their particular atmospheres for 48 and 72 h, respectively. Five samples (replicates) of infested fruit were prepared for each treatment. Each replicate consisted of 15–30 blueberries, 15 cherries, or 6 raspberries. The experiment was repeated twice.

#### Experiments Separately Assessing Effect of Elevated CO_2_ Concentration and Long-term Cold Storage

Since we saw that cold storage reduces the insecticidal effect of CO_2_ fumigation, a second set of experiments was performed where the CO_2_ fumigation was performed at room temperature, prior to cold storage. In these experiments, we used a shorter fumigation period (0, 1, 3, 5, and 8 h) so that the fruits’ shelf quality was not significantly reduced and was suitable for further storage. Within this timeframe, growers usually deliver the fruit from the orchard to storage after harvesting. In these experiments, only normal atmosphere (0.4%) and 100% CO_2_ treatment was performed. The fumigation was followed by 186 h cold storage. This resulted in 10 different treatments: 5 CO_2_ fumigation times at room temperature and 2 cold storage regimes—0 and 168 h cold storage. Five samples (replicates) of infested fruit were prepared for each treatment. Each replicate consisted of 20 blueberries. The experiment was repeated 3 times.

#### Verification Experiment: Effect of Elevated CO_2_ Concentration and Cold Storage on the Natural SWD Infestation on Raspberries

An experiment was conducted with naturally infested raspberries from orchards, to verify our results from experiments using fruit infested with laboratory reared *D. suzukii*. Raspberries (*Rubus idaeus* cv. ‘Amira’) naturally infested with *Drosophila suzukii* were produced under integrated production guidelines in the orchard of the Agricultural Institute of Slovenia at Brdo pri Lukovici (46°10ʹ02.4″ N, 14°40ʹ48.9″ E) where the presence of SWD population was confirmed by monitoring with food traps. The raspberries (1 kg) were harvested at the technological maturity stage and transported to the laboratory on the same day. Raspberry fruits were counted, weighed, and randomly divided into smaller samples of 15 raspberries (~30 g). Samples were transferred to plastic containers (insect breeding dish, square, 72 × 72 × 100 mm, HiMedia, India) with ventilation holes and nylon net on the lid to allow air circulation. Five samples (replicates) of infested fruit were prepared for each of the following treatments: (1) Control—naturally infested raspberries without CO_2_ and without cold storage were transferred directly into a growth chamber to assess the rate of natural infestation of SWD population (incubation conditions were the same as during rearing; see below, section ‘Procedures common to all experiments’); (2) 5hCO_2_—naturally infested raspberries were stored in 100 % CO_2_ at 21°C for 5 h and then transferred directly to a growth chamber for a 14-d incubation; (3) 5hCO_2_, chill—naturally infested raspberries were stored in 100% CO_2_ at 21°C for 5 h and then transferred directly to cold storage (4°C) for 1 wk. After 1 wk of cold storage, fruit samples were transferred to a growth chamber for 14 d; and (4) no CO_2_, chill—naturally infested raspberries were stored directly in cold storage for 1 wk in natural atmosphere (without elevated CO_2_). After 1 wk, the fruits were transferred to a growth chamber for a 14-d incubation.

#### Experiments Assessing Fruit Suitability for SWD Development

SWD-infested blueberries, cherries, and raspberries exposed to normal atmosphere and not subjected to cold storage were used in experiments assessing fruit species suitability for SWD development. The fruits were counted and weighed prior to incubation in growth chambers allowing us to express SWD-fruit infestation per number of fruits (e.g., flies per fruit) or per fruit mass (e.g., flies per g of fruit).

#### Procedures Common to All Experiments

A gas analyser (Geosensor-G100; Geotechnical Instruments Ltd, Coventry, UK) was used to determine the CO_2_ content in the polyethylene vacuum bags, and the temperature in the cold storage was constantly monitored (ThermaData Humidity-Temperature Logger, ETI ltd., UK).

After elevated CO_2_ exposure and/or cold storage (Aims 1, 2, and 3), or immediately after SWD infestation (Aim 4), the fruit samples were removed from the plastic bags and placed in a ventilated climate chamber with normal atmosphere and the same environmental conditions as during fly rearing. These conditions were maintained for 2 wk to allow the SWD eggs infesting the fruit to hatch, and SWD to complete their lifecycle and reach adulthood. After 14 d, the number of SWD flies in each sample was counted.

#### Statistical Data Analysis

The data were analyzed by general linear model (GLM), where the CO_2_ concentration, time of CO_2_ incubation before cold storage, fruit type or variety, and time of cold storage were considered fixed factors. Also, experiment repetition and interactions between the fixed factors were analyzed for their effect on the number of developed SWD. Tukey’s honestly significant difference (HSD) procedure at 95% confidence level was used to separate individual treatments based on their efficacy to hinder SWD development. The difference was considered significant at *P* < 0.05. If not stated otherwise, data presented are mean values ± standard error (SE). The number of biological replicates (*n*) is indicated in the figure or table captions. The analyses were performed with the statistical software Statgraphics Centurion XVI and XVIII (StatPoint Technologies, Inc., The Plains, VA, USA) and GraphPad Prism 5.00 (GraphPad Software, Inc., La Jolla, CA, USA).

## Results

The measured CO_2_ concentration of the atmosphere in the vacuum bags where 100% CO_2_ was desired contained 94.5 ± 0.4% CO_2_, whereas the vacuum bags where 10% CO_2_ was desired contained 11.2 ± 0.1% CO_2_. The temperature in the cold storage was 4.9 ± 0.1°C, with a minimum of 4.1°C and maximum of 7.3°C.

### CO_2_ and Cold Storage Negatively Affect SWD Development in Infested Fruit

#### Blueberries

**Fig. 1.**
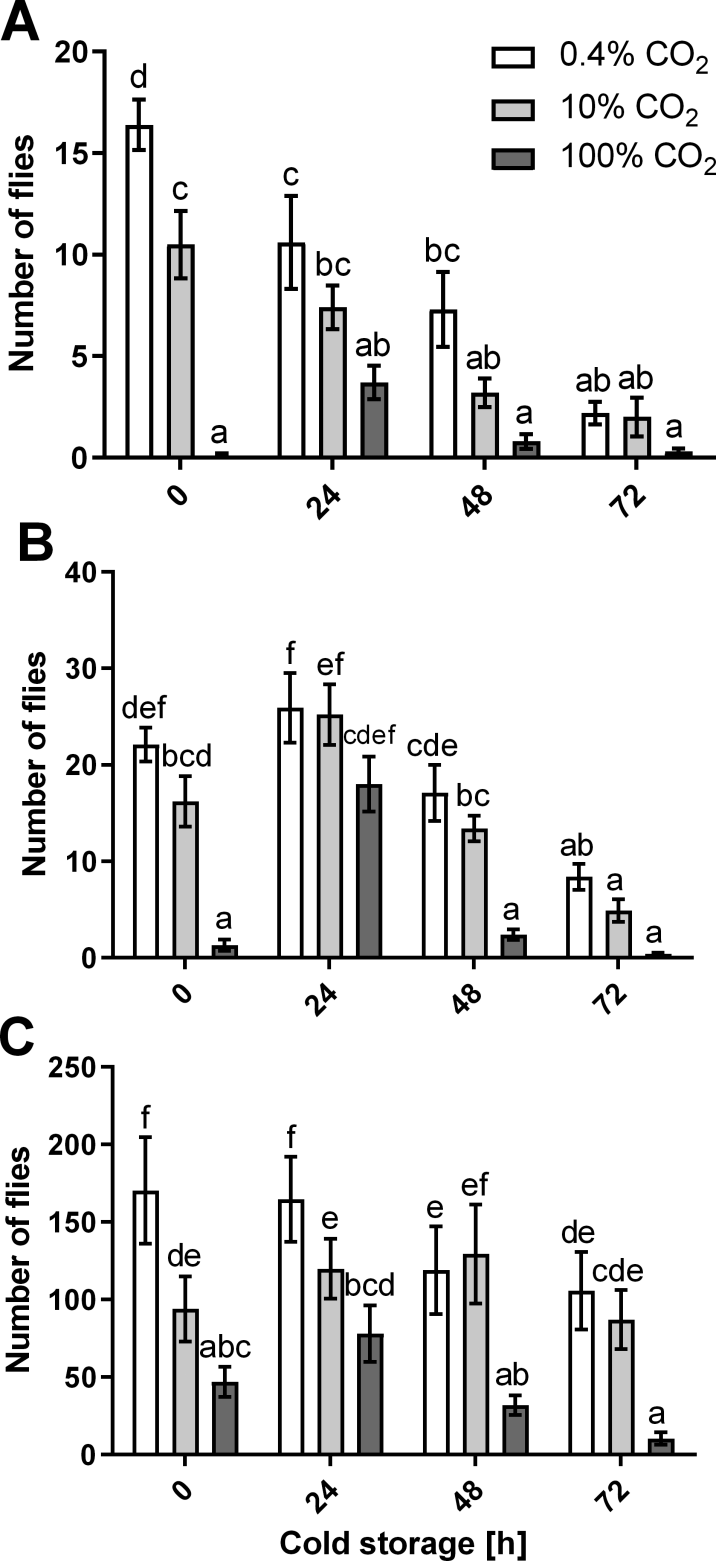
Effect of different concentrations of CO2 and different times of cold storage on the survival and development of Drosophila suzukii in blueberries (A), cherries (B) and raspberries (C). Data presented are averages ± standard error from 2 pooled experiments, each performed with 5 replicates per treatment (*n* = 10). Bars not sharing the same lower-case letter(s) are significantly different (*P* < 0.05) according to Tukey’s HSD post-test.

The highest number of SWD developed in the control treatment (no cold storage, normal air), where 16.4 ± 1.2 SWD flies developed on average, followed by those exposed to normal air and in cold storage for 24 h (10.6 ± 2.3 flies) and those exposed to 10% CO_2_ for 24 h without cold storage (10.5 ± 1.7). The lowest number of SWD developed in fruit incubated for 24 h in 100% CO_2_ at room temperature (0.1 ± 0.1), followed by fruit in cold storage and exposed to 100% CO_2_ for 72 h (0.3 ± 0.2) (Fig. 1A). The factors cold storage duration (*F* = 25.5; df = 3, 119; *P* < 0.0001), CO_2_ concentration (*F* = 45.1; df = 2, 119; *P* < 0.0001), and their interaction (*F* = 6.9; df = 6, 119; *P* < 0.0001), but not experiment repetition (*F* = 0.05; df = 1, 119; *P* < 0.0001), significantly affected the number of SWD developed in the (un)treated fruit.

### Cherries

The highest number of SWD developed in fruit in cold storage for 24 h in normal air (25.9 ± 3.6 SWD flies developed) and those exposed to 10% CO_2_ and in cold storage for 24 h (25.2 ± 3.1 flies). The lowest number of SWD developed in fruit in cold storage and exposed to 100% CO_2_ for 72 h (0.4 ± 0.2), followed by fruit incubated for 24 h in 100% CO_2_ at room temperature (1.3 ± 0.6) (Fig. 1B). 22.1 ± 1.8 SWD developed on average in the control treatment (no cold storage, normal air) (Fig. 1B). The factors cold storage duration (*F* = 47.4; df = 3, 119; *P* < 0.0001), CO_2_ concentration (*F* = 47.7; df = 2, 119; *P* < 0.0001), and their interaction (*F* = 2.8; df = 6, 119; *P* = 0.0131), as well as experiment repetition (*F* = 26.1; df = 1, 119; *P* < 0.0001), significantly affected the number of SWD developed in the (un)treated fruit.

#### Raspberries

The highest number of SWD developed in the control treatment (no cold storage, normal air), where 170 ± 34.4 SWD flies developed on average, followed by those exposed to normal air and in cold storage for 24 h (164 ± 27.3 flies) and those exposed to 10% CO_2_ and in cold storage for 48 h (129 ± 32.0). The lowest number of SWD developed in fruit in cold storage and exposed to 100% CO_2_ for 72 h (10.6 ± 3.9), followed by fruit incubated in 100% CO_2_ and in cold storage for 48 h (32.0 ± 6.2) (Fig. 1C). The factors cold storage duration (*F* = 8.5; df = 3, 117; *P* < 0.0001), CO_2_ concentration (*F* = 66.2; df = 2, 117; *P* < 0.0001), and their interaction (*F* = 2.4; df = 6, 117; *P* = 0.0326), as well as experiment repetition (*F* = 238; df = 1, 117; *P* < 0.0001), significantly affected the number of SWD developed in the (un)treated fruit.

### A Short Period of CO_2_ Treatment at Ambient Temperature Before Cold Storage Ensures Optimal Fruit Sanitation Effect

**Fig. 2.**
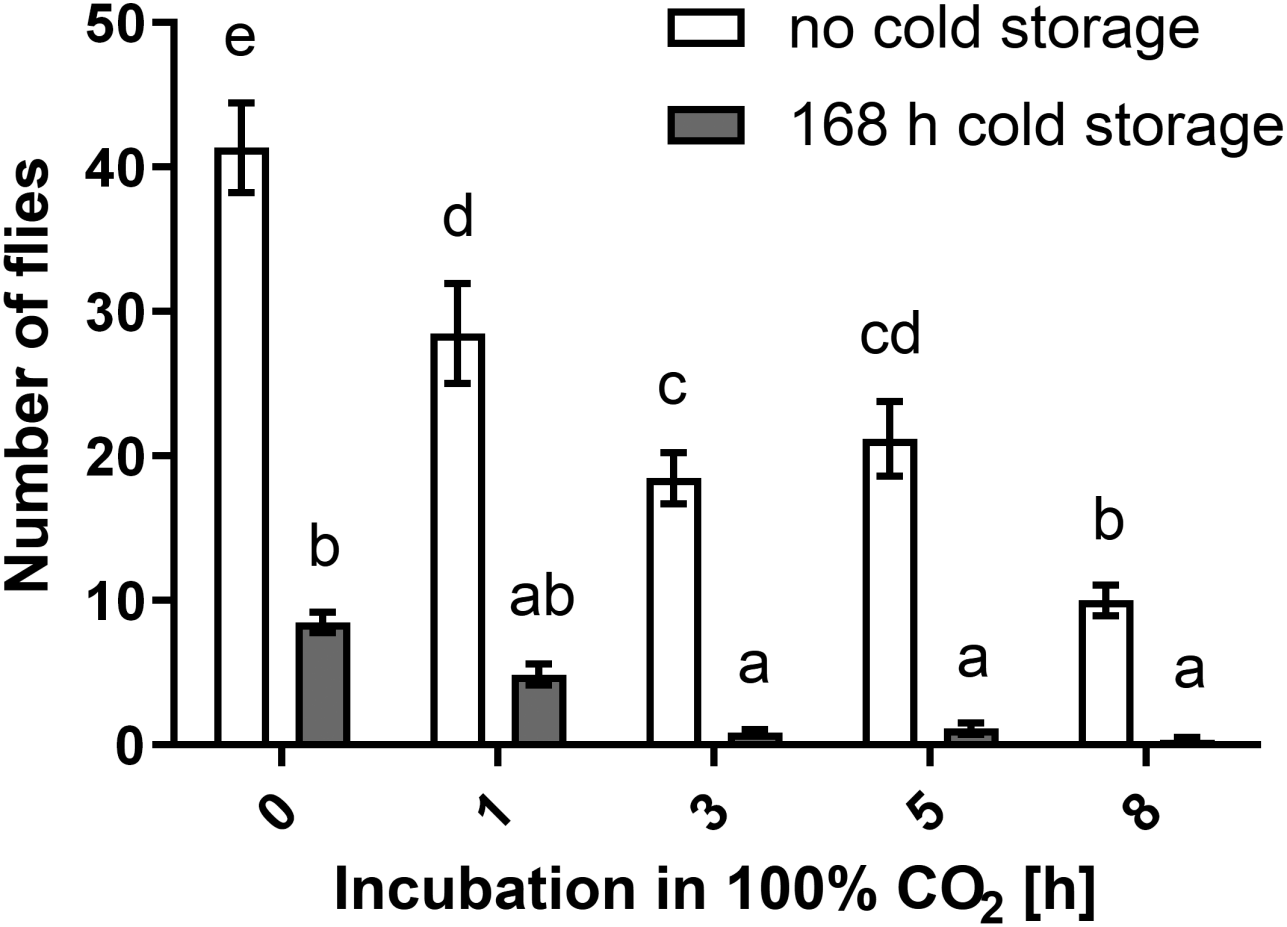
Effect of different times of fumigation of infested blueberries in a 100% CO2 atmosphere at ambient temperature and effect of cold storage on survival and development of *Drosophila suzukii*. Data presented are average ± standard error from 3 pooled experiments (*n* = 15). Bars not sharing the same lower-case letter(s) are significantly different (*P* < 0.05) according to Tukey’s HSD post-test.

The highest number of SWD developed in the control treatment (no cold storage, normal air), where 41.3 ± 3.8 SWD flies developed on average, followed by those exposed to 100% CO_2_ for 1 h without cold storage (28.5 ± 4 flies) and those exposed to 100% CO_2_ for 5 h without cold storage (21.0 ± 3.2). The lowest number of SWD developed in fruit incubated for 8 h in 100% CO_2_ prior to 168 h cold storage (0.3 ± 0.2), followed by fruit incubated for 3 h in 100% CO_2_ prior to 168 h cold storage (Fig. 2). The factors cold storage (*F* = 319; df = 1, 140; *P* < 0.0001), 100% CO_2_ fumigation time prior to cold storage (*F* = 34; df = 4, 140; *P* < 0.0001), and their interaction (*F* = 11; df = 4, 140; *P* < 0.0001), as well as experiment repetition (*F* = 15; df = 2, 140; *P* < 0.0001) significantly affected the number of SWD developed in the (un)treated fruit.

### Verification Experiment Confirmed the Effects of Elevated CO_2_ Concentration and Chilling on the Survival of Natural Population of the SWD

**Fig. 3.**
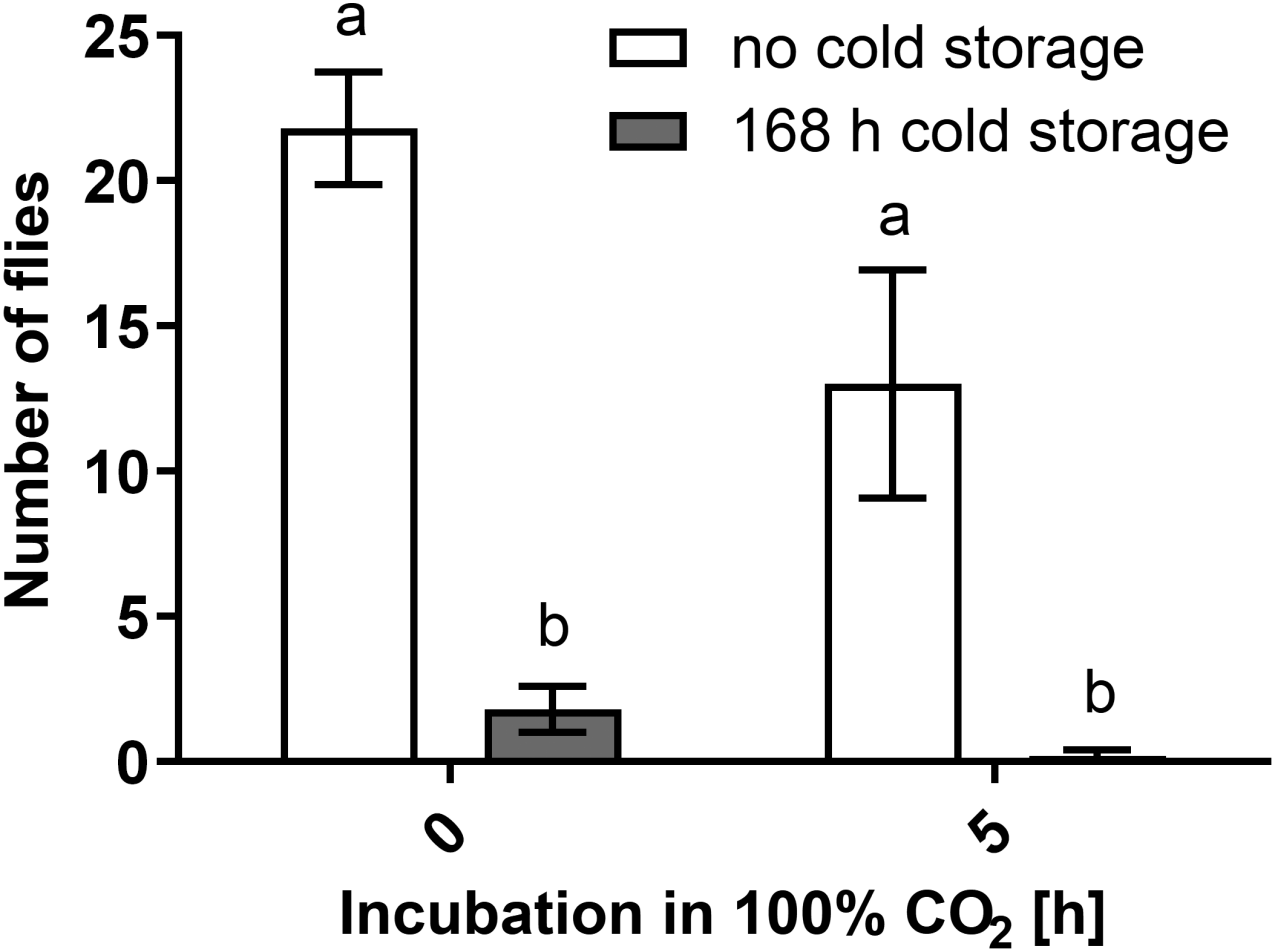
Average number of flies developed in various treatments in the verification experiment. Results presented are average values ± standard error, calculated from 5 replicates per treatment. The experiment was performed once (*n* = 5). Bars not sharing the same lower-case letter(s) are significantly different (*P* < 0.05) according to Tukey’s HSD post-test.

The highest number of SWD developed in the control treatment (no cold storage, normal air), where an average of 21.8 ± 1.9 flies developed, followed by those exposed to 100% CO_2_ for 5 h without cold storage (13.0 ± 3.9). The lowest number of SWD developed in fruit exposed to 100% CO_2_ for 5 h and chilled for 1 wk (0.2 ± 0.2), followed by fruit incubated in normal atmosphere and cold storage for 1 wk (1.8 ± 0.8) (Fig. 3).

### Raspberry Seems to be Most Suitable for SWD Development

The untreated, artificially infested fruits of the 3 investigated fruit species gave rise to different numbers of SWD. The highest number of SWD developed in raspberries (variety Amira; 95% confidence interval: 4.50–6.85 SWD g^−1^ fruit), followed by blueberries of variety Eliot (0.20–2.54) and Bluecrop (0.00–1.90). The fewest SWD developed in cherries (variety Kordia; 0.00–1.28) (Table 1).

**Table 1.** *Drosophila suzukii* infestation of and development success in different fruit species and varieties. Artificially infested fruit was incubated in normal air at 22°C and 70% RH for 14 d. Data presented are average ± standard error from 2 pooled experiments, each performed with 5 replicates per treatment (*n* = 10). Data not followed by the same lower-case letter(s) are significantly different (*P* < 0.05) according to Tukey’s HSD post-test.

Fruit	Variety	Flies per berry	Flies per g^−1^ fruit
**Cherry**	Kordia	1.47 ± 0.12a	0.11 ± 0.01a
**Blueberry**	Bluecrop	1.09 ± 0.08a	0.73 ± 0.06a
	Eliot	2.75 ± 0.28a	1.37 ± 0.14a
**Raspberry**	Amira	28.39 ± 5.73b	5.68 ± 1.15b

## Discussion

In the initial experiments, we found that elevated CO_2_ concentrations and cold storage both adversely impact SWD development in infested fruit. Subsequent experiments revealed that simultaneous low temperature treatment diminishes the insecticidal efficacy of CO_2_ fumigation. Therefore, a brief (3 h) 100% CO_2_ treatment at ambient temperature prior to cold storage was identified as means to ensure optimal fruit sanitation. Lastly, among various fruits not exposed to elevated CO_2_ or cold storage, raspberry emerged as the most suitable host for SWD development.

Our findings provide robust support for implementing cold storage as a postharvest treatment to mitigate the risk of live SWD infestations in soft fruits and cherries. The substantial reduction in the number of flies observed after 72 h at 4°C strongly implies the susceptibility of SWD eggs and larvae to extended periods in a cold environment. Typically, berry crops are stored at lower temperatures following harvest to extend shelf life and facilitate transportation, as noted in previous research (Terry et al. 2009). Moreover, maintaining storage conditions at or slightly above 0°C has proven effective in controlling various insects, particularly fruit flies, with a specific focus on SWD (Alonso et al. 2005, De Lima et al. 2011, Aly et al. 2017). It is noteworthy that the efficacy of cold treatment in reducing SWD damage increases exponentially with decreasing temperature. Prolonged exposure to cold conditions may significantly impact larval development time and decrease their overall survival rate, as highlighted in previous studies (Aly et al. 2017, Kim et al. 2018, Kraft et al. 2020).

Controlled atmospheres, such as elevated CO_2_ concentrations, offer an effective preservation technique to mitigate produce deterioration and slow maturation and senescence (Terry et al. 2009). They serve as an alternative or supplement to other disinfestation methods, including cold storage treatments, for arthropod infestation prevention (Mitcham et al. 2006). The specific effects of higher CO_2_ concentrations on insects depend on exposure duration and concentration (Mitcham et al. 1997, Riudavets et al. 2016). Follett et al. (2018) suggested that a 30-min exposure to 100% CO_2_ results in the death of all SWD larvae. In our study, CO_2_ significantly reduced adult SWD emergence, with 100% CO_2_ outperforming the 10% CO_2_ treatment. The hypothesis of postharvest treatments under controlled atmosphere posits that stressor accumulation leads to higher mortality. However, biochemical interactions between altered gas conditions and low temperature can act synergistically or antagonistically, and depending on the dynamics of the interaction, can either lead to a form of cross-tolerance that promotes pest survival or to increased pest mortality (Boardman et al. 2011). Gas treatments at low temperatures generally enhance pest mortality and reduce treatment duration (Alonso et al. 2005, Riudavets et al. 2016). Mostafa et al. (2021) reported consistent inhibition of SWD larvae growth and increased mortality rate after a 24-h exposure to 50% CO_2_ at 4°C for 48 h. In our study, incubating infested fruit in 10% CO_2_ with cold storage had no additional effect on SWD development, indicating its ineffectiveness. Conversely, a 24-h exposure to 100% CO_2_ at room temperature prevented almost all flies’ development in blueberries and cherries. Prolonged cold storage (e.g., 72 h) with 24-h 100% CO_2_ exposure had a synergistic effect, resulting in fewer flies compared to 72 h of cold storage or 100% CO_2_ alone.

Cold temperatures can counteract the adverse effects of oxygen deprivation by suppressing metabolic activities in ectothermic organisms (Boardman et al. 2016, Tarapacki et al. 2021). If this is applied to our study—combining CO_2_ fumigation with cold storage can nullify the fumigation effect. In sweet cherries, simultaneous cold storage and 100% CO_2_ exposure for 24 h resulted in an antagonistic interaction, leading to more fly development compared to cherries solely fumigated with 100% CO_2_ for 24 h. A similar, though insignificant, cold storage-fumigation interaction was observed in blueberries and raspberries. The findings indicate that cold storage diminishes potentially deleterious larval responses to CO_2_ by reducing the insects’ metabolic rate. High CO_2_ directly affects the heart and nervous system, decreasing pH, and anaerobic processes, impairing antioxidant response and membrane functioning. Survival reduction in combined treatment in a modified atmosphere and low temperature treatments may be attributed to one or a combination of these factors (Boardman et al. 2011). Prior research, as demonstrated in *D. melanogaster* and the larvae of the false codling moth (*Thaumatotibia leucotreta*), underscores the interactive effect between cold and anoxia. For instance, in *D. melanogaster*, when anoxia was administered at 3°C, the recovery time was significantly shorter compared to anoxia at 23°C, indicating a higher probability of fly survival at 3°C in anoxic conditions (Benasayag-Meszaros et al. 2015). Cross-tolerance between hypoxia and cold is well documented in *T. leucotreta* larvae, suggesting that cold enhances anoxia tolerance by reducing anaerobic metabolism and delaying detrimental effects (Boardman et al. 2016). Our initial findings demonstrated the potent insecticidal effect of 100% CO_2_ fumigation, prompting further experiments on blueberries with shorter exposures. Recognizing the industry’s need for swift fruit transfer to cold storage, we tailored the treatment for seamless integration into commercial production systems. A 3-h exposure to 100% CO_2_ at room temperature, followed by 1 wk of cold storage nearly halted SWD development, contrasting with some development observed after 1 h of incubation at room temperature followed by cold storage. Building on Mitcham et al’.s (1997) insight into slowed arthropod metabolism at lower temperatures, we hypothesized that the synergistic efficacy of high CO_2_ treatment would be enhanced with a room temperature fumigation followed by cold storage. The accelerated metabolism of pests at room temperature might exacerbate the impact of 100% CO_2_, making subsequent cold storage more effective in pest infestation mitigation. Considering that up to a 3-h exposure to room temperature has minimal impact on blueberry quality and shelf life if followed by cold storage (Boyette et al. 1993) our results, in line with existing studies, suggest advising producers to expose freshly picked blueberries to pure CO_2_ during transport to cold storage. This implementation, feasible through adapted transport boxes or field storage containers, could benefit both large professional producers and smaller soft fruit producers.

The results and trends of the verification experiment were directly comparable to the results obtained using laboratory-reared SWD population (focus on the bars above “0 h” and “5 h” in Figs 2 and 3). Regarding the natural SWD infestation, the majority of the emerging flies were identified as SWD, while only 3.26% (6 out of 184 flies) belonged to other Drosophilae species. Thus, the results of the verification experiment (natural infestation of SWD) confirmed the effects of elevated CO_2_ concentration and chilling on the survival of the laboratory reared SWD population in raspberries.

The 3 tested fruits exhibited varying susceptibility to SWD infestation in no-choice infestation experiments, with raspberries being the most susceptible, followed by blueberries and cherries. This is in sync with previous research which indicates that SWD favors soft-skinned and fleshy fruits, with raspberries and blackberries being particularly vulnerable (Lee et al. 2011, Walsh et al. 2011, Bellamy et al. 2013, Burrack et al. 2013, Abraham et al. 2015, Diepenbrock et al. 2016). Bioassays revealed a significant preference of adult flies for raspberries over other soft-skinned fruits (Bellamy et al. 2013). In orchards with diverse fruit species, raspberries experienced the highest larval infestation (Burrack et al. 2013). Furthermore, 2014 estimates for raspberry yield losses in North Carolina reported the highest damage, with an average of 41% crop loss due to SWD (Burrack 2014).

Most host susceptibility studies (e.g., Burrack et al. 2013, Arnó et al. 2016, Lee et al. 2016, Smrke et al. 2024) focused on fruit characteristics like sugar content, pH value, or firmness. Meanwhile, investigations into host preference, examining behaviors related to host selection (location, distribution, and frequency of hosts), are explored in other studies (e.g., Bellamy et al. 2013, Lee et al. 2016) which found that the probability of oviposition on a fruit increases as its penetrating force decreases and pH increases. Furthermore, yeasts play a crucial role in *Drosophila* larvae diet, impacting development and reproduction, as highlighted by Bellutti et al. (2018) and Plantamp et al. (2017). Jones et al. (2022) showed that raspberries have a higher Saccharomycetales yeast community than cherries during ripening. Consistent with Aly et al. (2017), Bellamy et al. (2013), and Burrack et al. (2013), raspberries proved to be the most suitable host for SWD in our study, with over a 5-fold higher SWD development in raspberries compared to blueberries and over 50 times more than in cherries.

## Conclusion

SWD can cause serious economic losses in soft and stone fruit production via in-field as well as postharvest fruit damage (Aly et al. 2017, Mazzi et al. 2017). This study focused on the latter aspect of the management strategy. We have shown that elevated CO_2_ concentrations alone or in concert with cold storage significantly reduce the number of SWD developed from artificially infested berries. The correct CO_2_ concentration and exposure time in combination with appropriate chilling period can be a straightforward postharvest SWD management strategy, attainable without sophisticated and expensive equipment. We have also shown that different fruit species, but not varieties investigated, are differentially susceptible to SWD infestation, or constitute a different reproductive environment for SWD. Future research should be aimed at (i) testing the presently developed SWD-infested fruit treatment protocol on other fruit species/varieties susceptible to SWD attack and (ii) developing an in-field CO_2_ fumigation apparatus, allowing fruit growers to treat their soft fruit en route to the cold-storage facilities.
